# Acetylcholinesterase Inhibitors Promote Angiogenesis in Chick Chorioallantoic Membrane and Inhibit Apoptosis of Endothelial Cells

**DOI:** 10.1155/2013/121068

**Published:** 2013-09-16

**Authors:** Seyed Mohsen Mortazavian, Heydar Parsaee, Seyed Hadi Mousavi, Zahra Tayarani-Najaran, Ahmad Ghorbani, Hamid Reza Sadeghnia

**Affiliations:** ^1^Department of Pharmacology, School of Medicine, Mashhad University of Medical Sciences, Mashhad 917794-8564, Iran; ^2^Pharmacological Research Center of Medicinal Plants, School of Medicine, Mashhad University of Medical Sciences, Mashhad 917794-8564, Iran; ^3^Department of Pharmacodynamics and Toxicology, School of Pharmacy, Mashhad University of Medical Sciences, Mashhad 91775-1365, Iran; ^4^Neurocognitive Research Center, School of Medicine, Mashhad University of Medical Sciences, Mashhad 917794-8564, Iran

## Abstract

Alzheimer's disease (AD) is one of the most common causes of dementia in the elderly. Recently, a great attention has been paid to the possible role of vascular changes in the pathogenesis of AD. Reduced microvascular density and degeneration of the endothelium are of structural cerebrovascular changes in AD. Acetylcholinesterase (AChE) inhibitors are widely used for the improvement of AD symptoms. Until now, however, the effects of AChE inhibitors on vascular changes including angiogenesis and endothelial cell apoptosis are not fully understood. In the present work, the effects of three AChE inhibitors (donepezil, rivastigmine, and galantamine) were tested on H_2_O_2_-induced apoptosis in human umbilical vein endothelial cells (HUVECs) and on angiogenesis in chicken chorioallantoic membrane model. Incubation of HUVEC with H_2_O_2_ led to a significant decrease in cell viability and an increase in the percentage of apoptotic cells. The tested drugs, at concentrations of 1–100 **μ**M, significantly inhibited the H_2_O_2_-induced toxicity. Also, all donepezil, rivastigmine and galantamine significantly increased the number of vessels in the chorioallantoic membrane when injected into fertilized eggs. In conclusion, AChE inhibitors possess angiogenesis-accelerating properties and have antiapoptotic effects on endothelial cells. These effects of AChE inhibitors may be involved in their beneficial effects on AD.

## 1. Introduction

Alzheimer's disease (AD), a progressive neurodegenerative disorder, is the primary common cause of dementia in the elderly. Intracellular neurofibrillary tangles, amyloid plaques, neuronal loss, and vascular amyloidosis are of characteristic hallmarks of AD [1]. In spite of extensive studies, however, the molecular pathogenesis of AD is not yet fully understood. Some of the suggested mechanisms include amyloid-induced neurotoxicity, inflammatory reaction, oxidative stress, proteasome inhibitor-induced cell death, and cerebral hypoperfusion [2, 3].

Recently, a great attention has been paid to the possible role of vascular changes in the pathogenesis of AD [1, 2, 4, 5]. Vascular irregularities, reduced microvascular density, arteriolar and capillary atrophy, and degeneration of the endothelium are of structural cerebrovascular changes in AD [6, 7]. Death or dysfunction of endothelial cells leads to dysregulation of endothelial-neuronal-glial cell interactions and therefore contributes to the onset or progression of this disease. During AD, endothelial cells can be damaged by glia-derived cytokines, heavily aggregated proteins, and oxidative stress [8].

Acetylcholinesterase (AChE) inhibitors, antiglutamic agents, secretase inhibitors, and anti-inflammatory drugs are of available therapeutics for management of AD [3, 9]. Cholinergic deficit is consistent and early finding in patient with AD and AChE inhibitors which increase bioavailability of ACh have proven to improve cognitive function in these patients [9–15]. Donepezil, rivastigmine and galantamine are of the most widely used AChE inhibitors, and currently approved by US Food and Drug Administration (FDA). Recent studies suggest that effects of AChE inhibitors are not limited to its cholinergic activity and other mechanisms including inhibition of glutamate-induced excitotoxicity, reducing inflammatory processes, and mitigating the effects of oxidative stress involved in their beneficial effects on AD [16, 17]. It has been well documented that overproduction of reactive oxygen cause dysfunction and death of endothelial cells [18]. However, the possible effect of AChE inhibitors on oxidative stress-induced endothelial cell damage remained unclear. Therefore, this study was carried out to investigate whether donepezil, rivastigmine, and galantamine are capable of protecting human umbilical vein endothelial cell (HUVEC) against H_2_O_2_-induced apoptosis, an *in vitro* model which simulates cell damage in AD [19, 20]. Also, the possible effect of these AChE inhibitors on angiogenesis was examined using chicken chorioallantoic membrane model. 

## 2. Materials and Methods

### 2.1. Drugs and Chemicals

The HUVECs were obtained from Pasteur Institute (Tehran, Iran). Dimethyl sulfoxide (DMSO), H_2_O_2_, penicillin/streptomycin solution, 3-(4,5-dimethyl-2-thiazolyl)-2,5-diphenyl-2H-tetrazolium bromide (MTT), and propidium iodide were purchased from Sigma (USA). Dulbecco's Modified Eagle's Medium (DMEM) and fetal bovine serum (FBS) were bought from GIBCO (USA). Fertilized eggs were kindly provided by Morghdaran Toos Co. (Mashhad, Iran). Rivastigmine tartrate and galantamine HBr (produced by Ranbaxy, India) were donated by Tehran Darou Co. (Iran). Donepezil HCl (produced by Megafine, India) was kindly provided by Razak pharmaceutical Co. (Iran).

### 2.2. Cell Cultures and Treatments

The HUVECs were cultivated in high-glucose DMEM supplemented with FBS (10%), penicillin (100 units/mL), and streptomycin (100 *μ*g/mL). All cells maintained in humidified atmosphere of 5% CO_2_ and 95% air at 37°C. Trypsin/EDTA solution was used to passage cells whenever they were grown to confluence. The cells at subconfluent stage were harvested from culture flask and seeded overnight in 96-well culture plate (15000 cells/well) after checking the viability with trypan blue exclusion technique. Then, to determine the effect of test drugs on cell viability, the medium was changed by a fresh one containing donepezil (0.1, 1, and 10 *μ*M), rivastigmine (1, 10, and100 *μ*M) and galantamine (1, 10, and 100 *μ*M) in the presence or absence of H_2_O_2_ (1 mM), and the cells were incubated for 24 h.

### 2.3. Cell Viability Assay

The viability of cells was determined using MTT colorimetric assay as described previously [21, 22]. Briefly, at the end of treatment, the MTT solution (5 mg/mL) was added to each well of culture plate with final concentration of 0.05%. After 4 h, the reaction mixture was removed, and the formazan precipitate was dissolved in DMSO. The optical density of formazan dye was read at 545 nm against 620 nm as background. The percentage of viable cells was calculated as the mean ± SEM with controls set to 100%. 

### 2.4. Cell-Cycle Analysis

The cells were seeded overnight in 12-well culture plate (75000 cells/well) and treated for 24 h with tested drugs. Then floating and adherent cells were harvested and incubated with 750 *μ*L of a hypotonic buffer (50 *μ*g/mL propidium iodide in 0.1% sodium citrate containing 0.1% triton X-100) at 4°C overnight in the dark [23]. Then, the cells were analyzed with a flow cytometer (BD FACSCanto, BD Sciences, San Jose, CA, USA), and the results were analyzed by WinMDI (version 2.8) software.

### 2.5. Chicken Chorioallantoic Membrane Angiogenesis Model

Fertilized eggs were incubated at 37°C and 70% relative humidity in a forced draught incubator. At day 8, a window opening is punctured on each egg and 0.01–10 nmol/egg of donepezil, rivastigmine, or galantamine was injected into the chorioallantoic sac. The control eggs received sterile PBS only. Then, the injection holes were closed by wax, and the eggs were maintained in the incubator. At day 12, the eggs were opened and the chorioallantoic membrane vasculatures were photographed using a stereo microscope equipped with a digital camera (Canon EOS 40D with Canon EF 100 mm f/2.8 USM macrolens). The angiogenic response was evaluated by counting the vessel density using Photoshop CS2 and image measurement software.

### 2.6. Statistical Analysis

Statistical comparison was made by one-way analysis of variance (ANOVA) followed by Tukey's post hoc test. Differences were considered significant when *P* values were less than 0.05. All experiments were performed three times in duplicate. The results were presented as the mean ± SD.

## 3. Results

### 3.1. Effect of AChE Inhibitors on Endothelial Cell Viability

**Figure 1:**
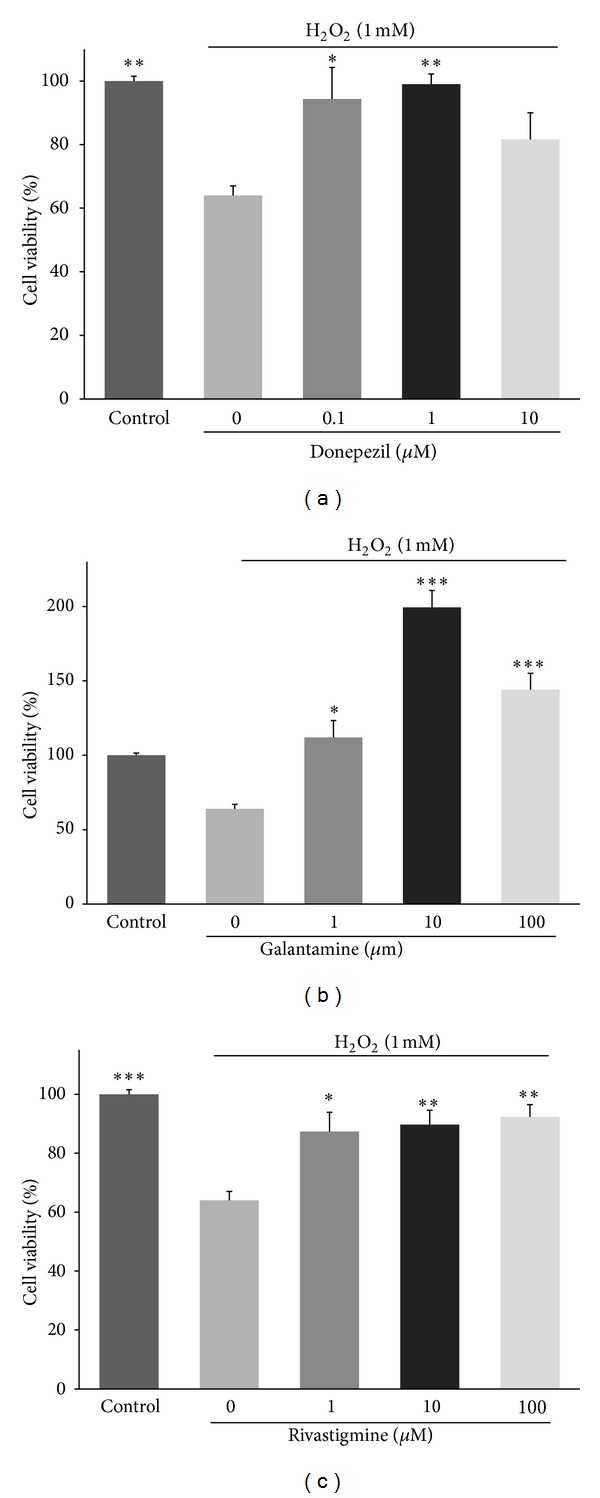
Effects of AChE inhibitors on viability of endothelial cells in the presence of H_2_O_2_. The HUVECs were seeded overnight in 96-well culture plate (5000 cells/well) and treated with donepezil, rivastigmine, and galantamine for 24 h. The percent of viability was normalized against untreated control cells. Data are mean ± SD of two independent experiments performed in triplicate. *P < 0.05, **P < 0.01, and ***P < 0.001 versus untreated cells cultured in the presence of H_2_O_2_.

Exposure of cultured HUVECs to H_2_O_2_ significantly decreased cell viability from 100 ± 3% (control) to 64 ± 5% (*P* < 0.001). Incubation with donepezil significantly attenuated the H_2_O_2_-induced cell death (Figure 1(a)). The percent of viability in the cells treated with 0.1 and 1 *μ*M of donepezil was 94 ± 17% (*P* < 0.05 versus untreated cells) and 99 ± 5% (*P* < 0.01), respectively. Also, the presence of 1, 10, and 100 *μ*M of galantamine in the cell medium increased the cell viability from 64 ± 5% (untreated cells) to 112 ± 19% (*P* < 0.05), 199 ± 19% (*P* < 0.001), and 144 ± 18% (*P* < 0.001), respectively, (Figure 1(b)). Similarly, the percent of viability in the cells treated with 1 *μ*M (87 ± 11%, *P* < 0.05), 10 *μ*M (90 ± 8%, *P* < 0.01), or 100 *μ*M (92 ± 7%, *P* < 0.01) of rivastigmine was significantly higher than that of untreated cells (Figure 1(c)).

### 3.2. Effect of AChE Inhibitors on Endothelial Cell Apoptosis

**Figure 2:**
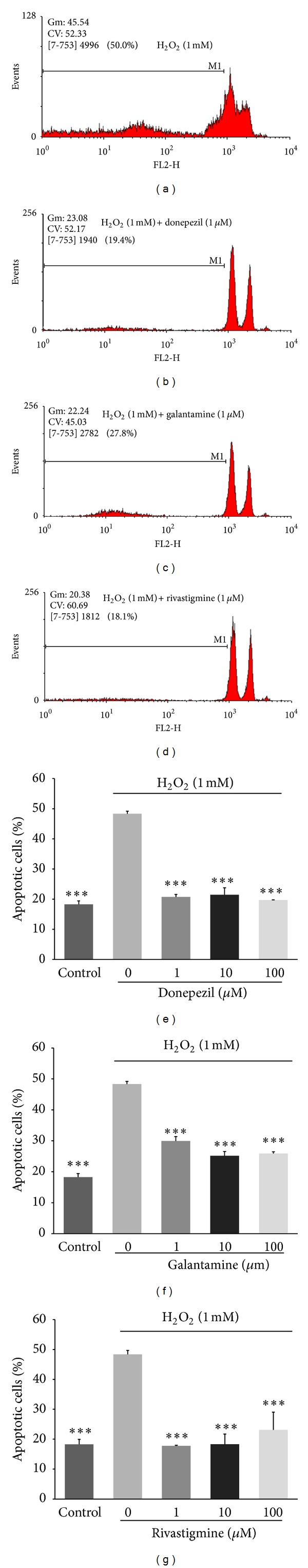
Effects of AChE inhibitors on H_2_O_2_-induced endothelial cells apoptosis. The HUVECs were seeded overnight in 12-well culture plate (50000 cells/well) and treated with donepezil, rivastigmine, and galantamine for 24 h. Data are mean ± SD of two independent experiments performed in triplicate. ***P < 0.001 versus untreated cells cultured in the presence of H_2_O_2_.

Flow cytometry revealed that in control condition, 18.3 ± 2.4% of HUVECs were in apoptosis stage. In the presence of H_2_O_2_, 48.3 ± 1.5% (*P* < 0.001 versus control) of cells were apoptotic (Figure 2(a)). The percentage of apoptotic cells significantly (*P* < 0.001) reduced by 1 *μ*M (20.8 ± 2%), 10 *μ*M (21.4 ± 4.8%), and 100 *μ*M (19.6 ± 0.1%) of donepezil (Figures 2(b) and 2(e)). Also, the percentage of apoptotic cells was significantly (*P* < 0.001) decreased in the cells treated with 1 *μ*M (30 ± 3%), 10 *μ*M (25 ± 2.8%), and 100 *μ*M (26 ± 1.1%) of galantamine (Figures 2(c) and 2(f)). Likewise, incubation with rivastigmine could reduce the H_2_O_2_-induced apoptosis (17.8 ± 0.6%, 18.5 ± 3.4%, and 23.2 ± 4.5% for 1, 10, and 100 *μ*M, resp., Figures 2(d) and 2(g)).

### 3.3. Effect of AChE Inhibitors on Angiogenesis

**Figure 3:**
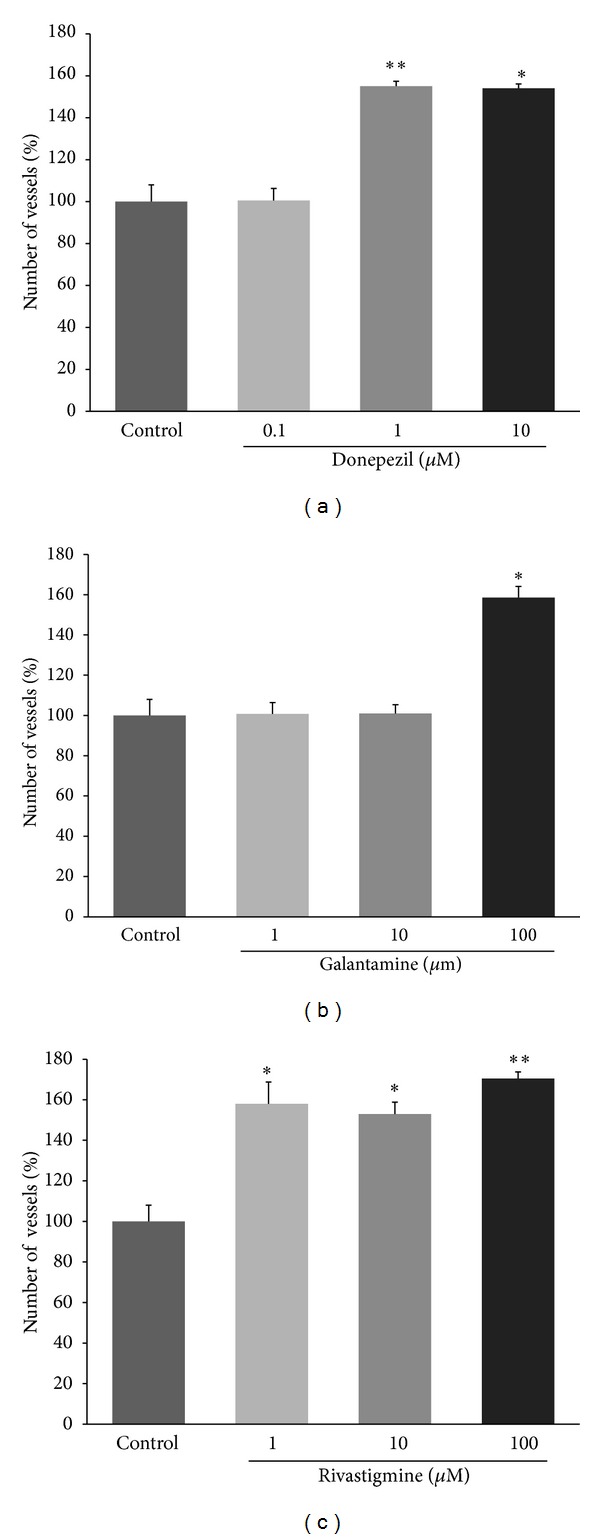
Effects of AChE inhibitors on angiogenesis in chicken chorioallantoic membrane model. The percent of vascularity was normalized against untreated control eggs. Data are mean ± SD of two independent experiments performed in triplicate. *P < 0.05, **P < 0.01, versus control.

**Figure 4:**
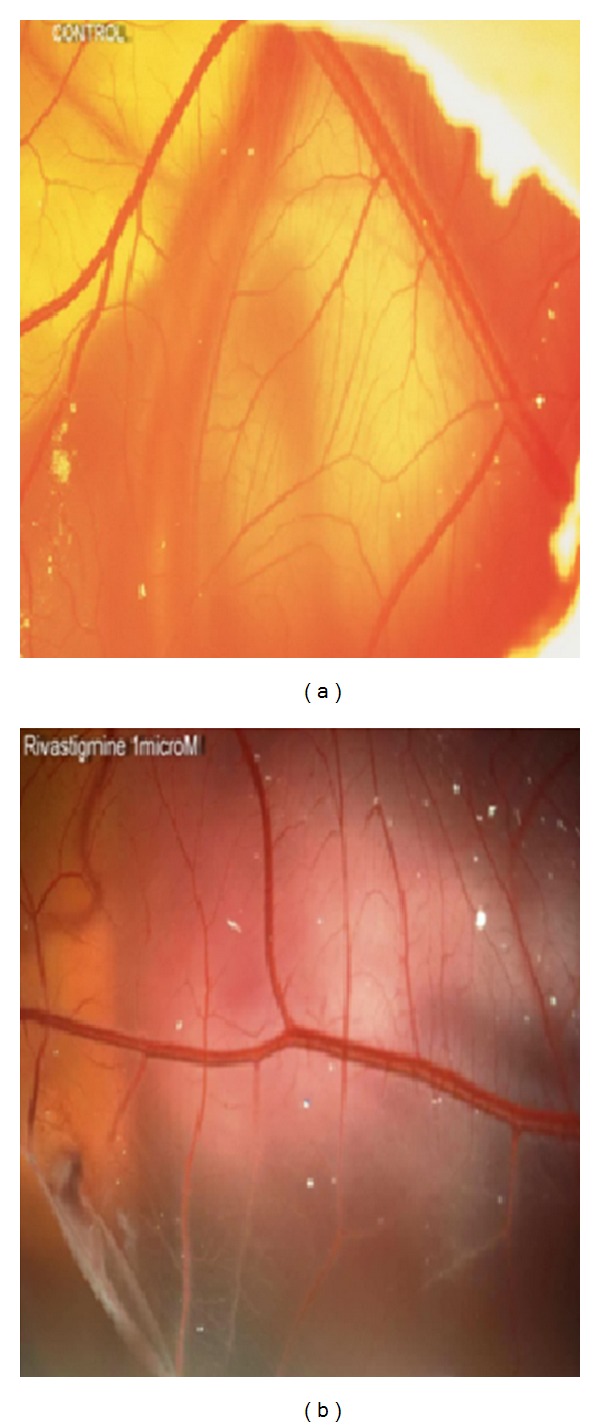
Effects of AChE inhibitors on angiogenesis in chicken chorioallantoic membrane model. Representative photographs of chorioallantoic membrane are shown for control (a) and rivastigmine (b) treated eggs.

Figure 3 shows the effect of ACE inhibitors on the number of vessels in chorioallantoic membrane. In the presence of 0.1 and 1 nmol/egg of donepezil, the number of vessels increased from 100±16% (control) to 155 ± 4% (*P* < 0.01) and 153 ± 4% (*P* < 0.05), respectively, (Figure 3(a)). Similarly, galantamine at 10 nmol/egg (158 ± 11%, *P* < 0.05) and rivastigmine at 0.1 nmol/egg (158 ± 23%, *P* < 0.05), 1 nmol/egg (153 ± 11%, *P* < 0.05), and 10 nmol/egg (170 ± 6%, *P* < 0.01) significantly increased angiogenesis (Figures 3(b) and 3(c)). The representative photographs of chorioallantoic membrane for control (PBS) and rivastigmine treated eggs are shown in Figure 4.

## 4. Discussion

AChE inhibitors are of the most currently used therapeutics for symptomatic improvement in AD [9]. The beneficial effects of AChE inhibitors are not limited to the enhancement of cholinergic activity. Other mechanisms such as inhibition of glutamate-induced neurotoxicity and reducing oxidative damage may also contribute [16, 17]. In the present study, we demonstrated that donepezil, rivastigmine, and galantamine are capable of protecting endothelial cells against oxidative stress-induced cytotoxicity. Incubation with these AChE inhibitors attenuated the H_2_O_2_-induced apoptosis and restored the proliferation of cultured endothelial cells. The cytoprotective effect was observed even at concentration of 0.1 and 1 *μ*M which is comparable to the steady-state plasma concentration of 0.1–0.3 *μ*M in patients that received different doses (10–24 mg/day) of donepezil, rivastigmine, and galantamine [24].

It is well documented that structural cerebrovascular changes (vascular irregularities, reduced microvascular density, arteriolar and capillary atrophy, and endothelium degeneration) are contributed to the pathogenesis of AD [6, 7]. Recent studies have shown that endothelial cells are target for the toxic effects of glia-derived cytokines, heavily aggregated proteins, and stimuli inducing oxidative stress in AD brains [8]. Oxidative stress plays a pivotal role in cerebrovascular dysfunction and endothelial cell apoptosis [25, 26]. Activation of apoptosis can occur primarily via intrinsic or extrinsic pathways. While the intrinsic pathway originates from mitochondrial release of cytochrome *c* and subsequent activation of caspase-3, the extrinsic pathway begins with a cell death receptor and associated stimulation of caspase-8 which in turn can activate caspase-3. Activated caspase-3 targets substrates that promote DNA fragmentation and cell death. H_2_O_2_ can activate both intrinsic and extrinsic apoptosis pathways in endothelial cells, leading to activation of caspases-3 and subsequent apoptosis [27]. Excess H_2_O_2_ disturbs balance between the production of reactive oxygen species and cellular antioxidant defenses (e.g., glutathione) and thereby damages macromolecules such as lipids, proteins, and nucleic acids, which trigger apoptotic pathways [28, 29]. 

Increased levels of H_2_O_2_ and lipid peroxidation have been shown to mediate amyloid beta neurotoxicity [19]. Also, oxidative stress is responsible for amyloid beta-induced cerebral angiopathy which is frequently observed in AD [26]. It has been shown that AChE inhibitors inhibit lipid peroxidation and increase glutathione level in the brain in the mice experimental model of dementia [30]. Therefore, these drugs, beside inhibition of acetylcholinesterase, may also mitigate the effects of oxidative stress on brain endothelial cell. The inhibitory effect of donepezil, rivastigmine, and galantamine on H_2_O_2_-induced apoptosis of endothelial cell may be involved in their beneficial effects on AD.

Our data also showed that donepezil, rivastigmine, and galantamine are able to increase angiogenesis. At present, however, the role of angiogenesis in pathogenesis of AD is not yet fully understood. Previous studies have shown that cognitive impairments in aging individuals are frequently associated with vascular diseases [31]. Also, conditions like atherosclerosis and diabetes which decrease vascular flow are associated with the increase of risk of AD. Decrease of vascular flow reduces amyloid-*β* efflux from brain and enhances angiogenesis. Deposition of amyloid beta accelerates the process of blood vessel branching. Over time, therefore, highly branched vessel networks appear in AD brain. While early angiogenesis restores blood flow and reduces amyloid beta, the resulting hypervascularization impairs perfusion efficiency [2, 5, 32, 33]. Thus, some scientists proposed that antiangiogenic drugs might be able to improve management of AD [2]. Recently, Miyazaki and colleagues reported that donepezil decreases vascular endothelial growth factor (VEGF) expression and attenuates angiogenesis in a mice hindlimb ischaemia model [34]. With the same model, however, Kakinuma and coworkers observed that donepezil enhances VEGF expression and promotes tube formation in endothelial cells [10]. In the present study, we showed that donepezil, rivastigmine, and galantamine could increase the number of vessels in chicken chorioallantoic membrane model. The proangiogenic effect was observed at dose of 0.01–10 nmol/egg which is a close approximation to the *in vivo* situation [24]. Although our data are in concert with the results of Kakinuma and coworkers, it is too early to draw any conclusion on the relationship between AChE inhibitors, angiogenesis, and AD. 

Taken together, in the present study, we demonstrated that donepezil, rivastigmine, and galantamine promoted angiogenesis and also protected endothelial cells against oxidative stress-induced cytotoxicity. Therefore, these effects of AChE inhibitors may be involved in their beneficial effects on AD.
